# Lymphocytes Mitochondrial Physiology as Biomarker of Energy Metabolism during Fasted and Fed Conditions

**DOI:** 10.1100/2012/629326

**Published:** 2012-03-12

**Authors:** Erika Cortez, Fabiana A. Neves, Amélia F. Bernardo, Ana Carolina Stumbo, Laís Carvalho, Érica Garcia-Souza, Rosely Sichieri, Anibal S. Moura

**Affiliations:** ^1^Laboratory of Physiology of Nutrition and Development, Department of Physiological Sciences, Institute of Biology, Rio de Janeiro State University, Avenue 28 de Setembro 87, 5th Floor, Vila Isabel, 20551-030 Rio de Janeiro, RJ, Brazil; ^2^Laboratory of Cell Culture, Department of Histology and Embryology, Institute of Biology, Rio de Janeiro State University, 20551-030 Rio de Janeiro, RJ, Brazil; ^3^Department of Epidemiology, Institute of Social Medicine, Rio de Janeiro State University, 20551-030 Rio de Janeiro, RJ, Brazil

## Abstract

Mitochondria are central coordinators of energy metabolism, and changes of their physiology have long been associated with metabolic disorders. Thus, observations of energy dynamics in different cell types are of utmost importance. Therefore, tools with quick and easy handling are needed for consistent evaluations of such interventions. In this paper, our main hypothesis is that during different nutritional situations lymphocytes mitochondrial physiology could be associated with the metabolism of other cell types, such as cardiomyocytes, and consequently be used as metabolic biomarker. Blood lymphocytes and heart muscle fibers were obtained from both fed and 24 h-fasted mice, and mitochondrial analysis was assessed by high-resolution respirometry and western blotting. Carbohydrate-linked oxidation and fatty acid oxidation were significantly higher after fasting. Carnitine palmitoil transferase 1 and uncouple protein 2 contents were increased in the fasted group, while the glucose transporters 1 and 4 and the ratio phosphorylated AMP-activated protein kinase/AMPK did not change between groups. In summary, under a nutritional status modification, mitochondria demonstrated earlier adaptive capacity than other metabolic sensors such as glucose transporters and AMPK, suggesting the accuracy of mitochondria physiology of lymphocytes as biomarker for metabolic changes.

## 1. Introduction

Body weight, waist-hip ratio, glycemia, and serum lipids are common endpoints of nutritional interventions that require long term followup. In addition, these parameters are of low effectiveness to understand, for example, physiological mechanisms underlying changes induced by nutritional interventions, which require accurate biomarkers in order to evaluate their efficacy [1].

 A biomarker is a measurable change related to a phenotype. A valid nutritional biomarker can also function as a key measure linking a specific exposure of a dietary compound to a health outcome and thus offers great potential to understand the relationship between diet and health. In summary, biomarkers are indicators of molecular and cellular events in biological systems and may help epidemiologists and clinicians better understand relationships between interventions, such as diets, and human health effects [2].

 It is not recently the discussion about the accuracy of biomarkers. Bistrian et al. [3, 4] demonstrated that the body mass index (BMI) did not allow detecting medical patient malnutrition. Moreover, McWhirter and Pennington [5] demonstrated that 40% of patients from a hospital school were undernourished and that it had not been early detected by the routinely used parameters. In another study about nutritional status, McWhirter et al. [6] showed that the BMI was not a good indicator to detect fat or muscle reduction, resulting in false-positive scores to naturally lean people.

 When considering storage complexity and energy expenditure that result in body weight modifications, variables such as age, gender, and health of patients (or population) arise a demand for markers which reflect the physiological complexity that interacts with nutrient availability [7, 8]. For example, individuals included in studies with random allocation, to evaluate dietary intervention and weight loss present higher response variability. Recently, our group tested a low-glycemic-index diet to improve weigh loss in insulin-resistant subjects, but the hypothesis was not confirmed by the commonly used biomarkers [9]. In other study, with teenagers, it was observed that BMI had low ability to predict dyslipidemia in this group [10].

 Thus, the characterization of biomarkers that allow measurement of intracellular energetic flux can make an important contribution in many fields and for diseases associated with the energetic balance. The option for mitochondrial activity study is of great interest due to its unique importance in the metabolism, quickly adaptation capacity to new effectors, and its relationship with different pathologies [11]. Oxidative phosphorylation (OXPHOS) is responsible for producing most of the ATP that is required by eukaryotic cells. Defects in OXPHOS encompass a large array of mitochondrial disorders with onset of clinical symptoms occurring at any age. As conclusive diagnostic evidence of an OXPHOS disorder often requires a muscle biopsy for enzyme analysis, it would be beneficial to have a less invasive and more accessible screening tool [12].

 Lymphocytes represent an easily obtainable source of tissue that presents advantages over the use of muscle biopsy. In addition, it was already demonstrated that mitochondrial physiology of lymphocytes is related to nutritional status and pathologies [13, 14]. On this way, we present the hypothesis that mitochondrial physiology of lymphocytes could be used as biomarker of nutritional states.

## 2. Experimental Methods

### 2.1. Animals

Three-month-old *Swiss* mice were provided by the Vital Brazil Institute (Rio de Janeiro, Brazil) and housed with three mice per cage in a temperature-controlled room and 12 h light/12 h dark cycle, with free access to water and standard laboratory chow. Mice were distributed into two groups: control group (CG, *n* = 8) and 24 h fasted group (FG, *n* = 8), in which animals were food private for 24 h, with free access to water. All experimental procedures were in accordance with institutional regulations for the care and use of laboratory animals.

### 2.2. Blood Lymphocytes Isolation

Mice were treated with heparin (1500 IU/kg body weight, i.v.) and anaesthetized with Avertin (2,2,2-tribromoethanol, 2-metil-2-buthanol; 0.02 mL/g body weight), chest was opened, and blood was collected. Samples were diluted with 0.9% NaCl (1 : 3 ratio) and submitted to a Ficoll-Hypaque (Sigma-Aldrich) density gradient. After centrifugation at 2000 rpm for 25 min, the mononuclear cells interface was collected, washed, plated in culture flasks with RPMI-1640 (Sigma Aldrich, St. Louis, MO, USA) plus FCS 10%, and incubated for 1 h at 37°C in a 5% CO_2_ atmosphere in air to allow monocytes adherence. Then, the supernatant containing 98% of lymphocytes was collected and used for the experiments.

### 2.3. Preparation of Skinned Muscle Fibers [15]

Mice were treated with heparin and anaesthetized as described above, chest was opened, and the heart, when still beating, excised and put into cooled relaxing and preservation solution, BIOPS (in mM: CaK2EGTA 2.77, K2EGTA 7.23, MgCl_2_ 6.56, dithiothreitol 0.5, K-MES 50, imidazole 20, taurine 20, Na2ATP 5.77, phosphocreatine 15, pH 7.1 adjusted at 25°C). Cooled hearts were cut into halves and muscle strips (2–4 mm long and 1–1.5 mm in diameter, 5–7 mg of wet weight) cut from myocardium of left ventricles along fiber orientation to avoid mechanical damage of the cells. By using sharpened forceps, the muscle fibers were separated from each other leaving only small areas of contact. After that, the fibers were transferred into vessels with cooled (in ice) BIOPS containing 50 *μ*g of saponin per mL and incubated at mild stirring for 30 min for complete solubilization of the sarcolemma. Permeabilized (skinned) fibers were then washed for 10 min in mitochondrial respiration medium, MIR05 (in mM: EGTA 0.5, MgCl_2_ 3.0, K-MES 60, taurine 20, K_2_HPO_4_ 10, HEPES 20, Sucrose 110 and BSA 1 g/L, pH 7.1 adjusted at 25°C).

### 2.4. High-Resolution Respirometry Protocol

Lymphocytes and skinned muscle fibers respiratory rates were determined with the Oroboros 2k-Oxygraph (Oroboros Instruments, Innsbruck, Austria) in 2 mL of MIR05 at 37°C with continuous stirring. Datlab software (Oroboros Instruments) was used for data acquisition and analysis. Before adding the cardiac tissue into oxygraph chamber, wet weight measurements were taken and a sample of 2-3 mg was used per chamber. Lymphocytes were counted in Neubauer chamber, and at least 10^6^ cells were necessary per chamber. Measurements of muscle fibers respiration were achieved at oxygen concentrations above 400 nmol·mL^−1^ in the chamber to prevent oxygen limitation. Oxygen consumption rates were expressed as pmol of O_2_·s^−1^·mg wet weight^−1^ for muscle fibers and pmol of O_2_·s^−1^·10^6^ cells^−1^ for lymphocytes. Digitonin was added to MIR05 in the chamber containing lymphocytes, in order to permeabilize the cells. The concentration of digitonin (2 *μ*M) was chosen out of a series of pilot experiments using a range (0–10 *μ*M) of concentrations.

Studies were performed with two sets of substrates. In the carbohydrate protocol, substrate combinations were used for electron flow through CI and CII (in mM): glutamate 10, malate 2, and succinate 10. In the fatty acid protocol, respiration was measured with (in mM) palmitoyl-L-carnitine 0.02 and malate 2. OXPHOS capacity is the oxygen consumption coupled to phosphorylation of ADP to ATP, determined after addition of saturating ADP concentration (5 mM), named as state 3. Oligomycin (1 *μ*g/mL), an ATP synthase inhibitor, was added for measurement of respiration rates in the absence of ADP phosphorylation, state 4, indicative of proton leak from the intermembrane space into mitochondrial matrix. From the respiratory fluxes obtained during the substrate titration protocol, respiratory control ratio (RCR) was calculated for state 3/state 4. Addition of cytochrome c (10 *μ*M) provided an evaluation of mitochondrial membrane integrity.

### 2.5. Western Blotting

Lymphocytes and fragments of myocardium were taken from the same samples used for respirometry and immediately frozen in liquid nitrogen. Carnitine palmitoil transferase 1 (CPT1), uncouple protein 2 (UCP2), insulin-independent glucose transporter SLC2A1 (GLUT1), insulin-dependent transporter SLC2A4 (GLUT4), AMP-activated protein (AMPK), and phosphorylated AMPK (p-AMPK) levels were determined by immunoblotting the extracts of lymphocytes and myocardium from the control and fasted groups. Samples were lysed in a buffer containing 50 mmol/L HEPES, 1 mmol/L MgCl_2_, 10 mmol/L EDTA, 1% Triton X-100, 10 mg/mL aprotinin, 10 mg/mL leupeptin, and 17.4 mg/mL PMSF (phenylmethanesulfonyl fluoride; Sigma-Aldrich). Homogenized samples were centrifuged for 15 min at 13000 rpm at 4°C, the supernatant was collected, and the total protein content was determined by the BCA method (Protein Assay Kit, Bicinchoninic Acid; Thermo scientific, Rockford, IL, USA). Samples (30 *μ*g) were run on 12% SDS-PAGE gels. The samples were loaded with a protein standard (Sigma-Aldrich) and then transferred to a PVDF Hybond-P membrane (Amersham, Buckinghamshire, UK), which was blocked with Tween-TBS (20 mM Tris-HCl, pH: 7.5; 500 mM NaCl; 0.01% Tween-20) containing 2% bovine serum albumin (Merck, Darmstadt, Germany). Primary antibodies used were rabbit anti-CPT1, anti-GLUT1, anti-GLUT4 (Santa Cruz Biotechnology, CA, USA), anti-AMPK*α* and anti-p-AMPK*α* (Cell Signaling Technology), anti-Actin (Sigma-Aldrich), and goat anti-UCP2 (Santa Cruz Biotechnology), all at a dilution of 1 : 1000. PVDF membranes were then incubated with an anti-rabbit or anti-goat secondary biotin-conjugated antibody (Santa Cruz Biotechnology), followed by incubation with horseradish peroxidase-conjugated streptavidin (Zymed Laboratories, INC., South San Francisco, CA, USA). Immunoreactive proteins were visualized using the ECL-Plus Western blotting detection kit (GE Healthcare, UK). The bands were quantified by densitometry using Image J Software (NIH, Bethesda, MD, USA). The relative gray level of target proteins over reference protein was used to represent the signal strength for each protein.

### 2.6. Statistical Analysis

The results were expressed as mean ± standard error of mean (SEM) of 8 animals per group, and statistical significance was assessed by Student *t*-test; *P* < 0.05 was regarded as statistically significant.

## 3. Results

### 3.1. Body Weight

The glycemia of FG was significantly decreased compared to CG after 24 h of fasting (*P* < 0.05). In addition, due to the prolonged fasting, FG had a reduction of 4.3% in body weight, which was not observed in the CG, but this difference was not statistically significant (Table 1).

### 3.2. High-Resolution Respirometry

**Figure 1:**
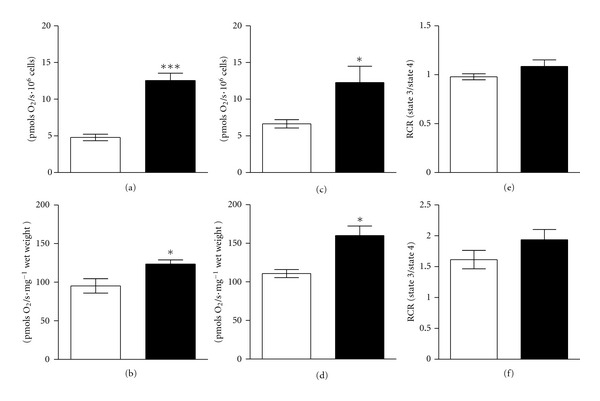
High-resolution respirometry of (a, c, e) lymphocytes and (b, d, f) muscle fibers obtained from control group (white bars) and 24 h-fasted group (black bars). Graphics show the maximally stimulated state 3 respiration during (a, b) fatty acid oxidation and (c, d) carbohydrate-linked oxidation. (e, f) Respiratory control ratio (RCR) calculated as state 3/state 4 respiration. Data are mean ± SEM. Asterisks denote significant difference (*P < 0.05 and ***P < 0.001) from values for control group as calculated by Student's *t*-test.

After fasting, the addition of carbohydrate-linked substrates or fatty acid to the oxygraph chamber significantly increased mitochondrial maximally ADP-stimulated state 3 respiration rates of both lymphocytes and muscle fibers compared to CG (Figure 1). The respiratory control ratio was not different between groups, despite of cell type, indicating that the prolonged fasting did not interfere with OXPHOS coupling (Figure 1). In all groups, the lack of a significant increase in respiration after addition of cytochrome c confirmed the integrity of the outer mitochondrial membrane (data not shown).

### 3.3. Western Blotting

**Figure 2:**
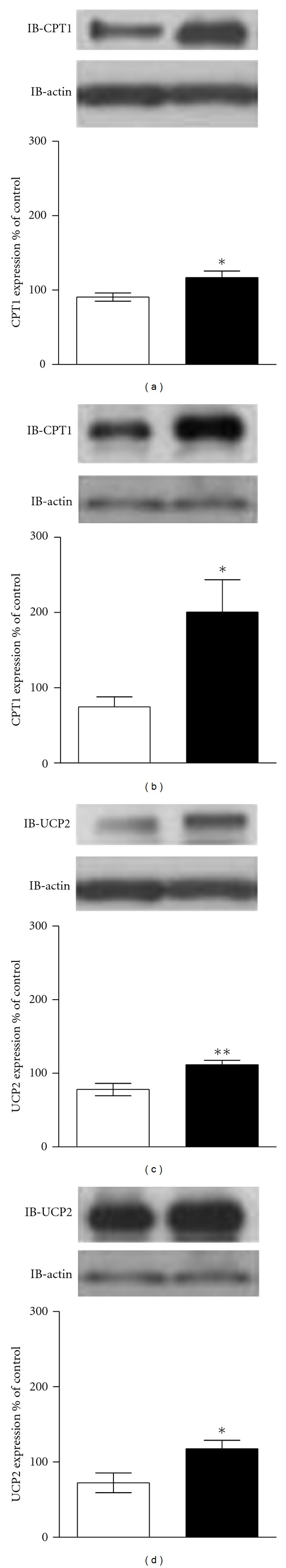
Western blotting analysis of CPT1 and UCP2 contents of (a, c) lymphocytes and (b, d) cardiomyocytes obtained from control group (white bars) and 24 h-fasted group (black bars). Graphics show the relative density of proteins levels which were normalized to those of Actin. Values represent mean ± SEM. Asterisks denote significant difference (*P < 0.05 and **P < 0.01) from values for control group as calculated by Student's *t*-test.

**Figure 3:**
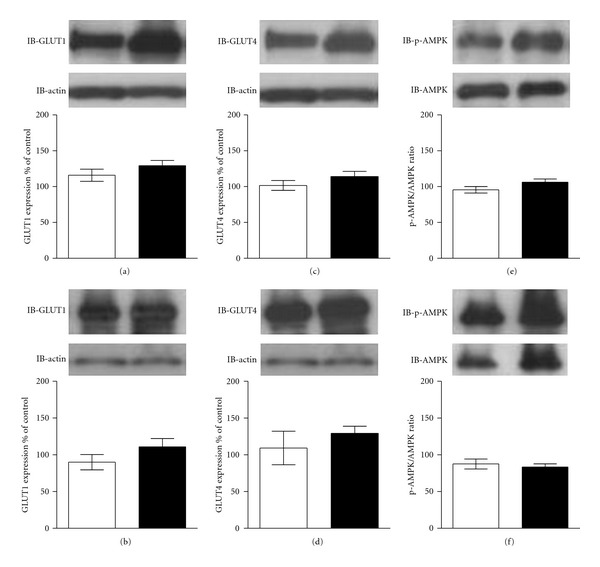
Western blotting analysis of GLUT1 and GLUT4 contents, and the pAMPK/AMPK ratio of (a, c, e) lymphocytes and (b, d, f) cardiomyocytes obtained from control group (white bars) and 24 h-fasted group (black bars). Graphics show the relative density of proteins levels which were normalized to those of Actin. Values represent mean ± SEM. Statistical analysis was calculated by Student's *t*-test.

The content of the mitochondrial proteins CPT1 and UCP2 was increased in FG of both lymphocytes (*P* < 0.05 and *P* < 0.01, resp.) and muscle fibers (*P* < 0.05) (Figure 2). On the other hand, the content of GLUT1 and GLUT4 and also the p-AMPK/AMPK ratio were not altered after fasting, regardless of cell type (Figure 3).

## 4. Discussion

It is well known that nutritional diseases such as obesity, hyperglycemia, and insulin resistance are associated with mitochondrial oxidative phosphorylation (OXPHOS). The effects of a hypocaloric diet on carbohydrate metabolism and OXPHOS of individuals initially diagnosed as insulin-resistant showed that body weight reduction was associated to OXPHOS increases during the postprandial period. On this way, OXPHOS is presented as a differentiated marker of insulin secretion concentration and capacity [16]. In another work, obese subjects submitted to hypocaloric diets demonstrated, after 8 weeks, reduction of inflammatory and oxidative stress markers and also of leptin levels, and an increase in leukocytes ATP contents [17].

 In addition to studies that show accuracy and correlation of the markers from mitochondrial metabolism and the response to nutritional interventions, it is also important to emphasize the speed as these markers are revealed. Complex I activity (the first enzyme of the electron transport chain) of peripheral blood mononuclear cells is reduced in response to malnutrition, but increases fast and is normalized one week after the onset of nutritional interventions, while the traditional markers of nutritional status do not do so [12, 18].

 Our data demonstrated increased carbohydrate-linked oxidation and fatty acid oxidation of lymphocytes and muscle fibers after 24 h of fasting, and also similar respiratory control ratios, indicating that lymphocyte OXPHOS correlates to that of muscle fibers after a nutritional status modification. This result is corroborated by Briet and Jeejeebhoy [19], which showed that the complex I activity in soleus muscle correlated with the complex I activity in mononuclear cells, in a study of the malnutrition and refeeding effect on mitochondrial enzyme activities.

 Western blotting data showed that lymphocytes and muscle fibers of FG presented significantly higher contents of the mitochondrial proteins CPT1 and UCP2 when compared to CG, which is consistent with the increased FA oxidation observed during fasting. It was already described that UCP2 gene expression increases in cardiomyocytes of 48 h-fasted mice [20]. GLUT1 and GLUT4 contents were unchanged between groups, regardless of cell type. Also, activated AMPK (p-AMPK) and the p-AMPK/AMPK ratio were not significantly different after 24 h fasting. These results showed that, under a nutritional status modification, mitochondria presented earlier adaptation capacity then other metabolic sensors such as glucose transporters and AMPK, confirming that mitochondrial physiology is a more accurate biomarker of nutritional state.

 Muscle biopsies have been used to study bioenergetics modification in different pathologies and nutritional interventions [21]. Lymphocytes may be particularly amenable to metabolic manipulation, demonstrate quickly nutritional modification [22], and also have the advantages of being easily obtained cells compared to muscle biopsies. So, we tested the hypothesis that under nutritional changes lymphocytes mitochondrial physiology is related to that of other cell types, such as cardiomyocytes, and thus could be used as biomarker of nutritional status. Using fasting as a nutritional intervention, we confirmed our hypothesis, demonstrating that lymphocytes present mitochondrial physiology modifications similar to that of muscle fibers. Due to the minimal invasiveness of blood collection, this method could provide an objective tool for monitoring response to nutritional interventions, treatments and evaluating progression of metabolic diseases. On this way, we conclude that mitochondrial physiology evaluation of circulating lymphocytes can be used as a reliable analysis of metabolism, contributing to implementation of a more accurate biomarker of nutritional interventions.

## Figures and Tables

**Table 1:** Glycemia, body weight at time zero, and body weight after 24 h of control and fasted mice. Data are mean ± SEM of 8 mice per group. Asterisks denote significant difference (*P < 0.05) from values for control group as calculated by Student's *t*-test.

Investigated Parameters	Glycemia (mg/dL)	Body weight *t *= 0 (g)	Body weight *t *= 24 h (g)
Control group (*n* = 8)	159.0 ± 13.6	53.43 ± 3.2	53.77 ± 3.3
Fasted group (*n* = 8)	104.7 ± 12.0*	53.03 ± 3.7	50.77 ± 4.8
